# Evolution of Bacterial Communities, Physicochemical Changes and Sensorial Attributes of Natural Whole and Cracked Picual Table Olives During Spontaneous and Inoculated Fermentation

**DOI:** 10.3389/fmicb.2020.01128

**Published:** 2020-05-29

**Authors:** Dimitrios A. Anagnostopoulos, Eleni Kamilari, Dimitrios Tsaltas

**Affiliations:** Department of Agricultural Sciences, Biotechnology and Food Science, Cyprus University of Technology, Limassol, Cyprus

**Keywords:** table olives, fermentation, Picual, microbial communities, metagenomics, organoleptic, physicochemical

## Abstract

Table olives are one of the most well-known traditionally fermented products, and their global consumption is exponentially increasing. In direct brining, table olives are produced spontaneously, without any debittering pre-treatment. Up to date, fermentation process remains empirical and inconstant, as it is affected by the physicochemical attributes of the fruit, tree and fruit management of pro and post-harvest. In the present study, whole and cracked Picual table olives were fermented at industrial scale for 120 days, using three distinct methods (natural fermentation, inoculation with lactic acid bacteria (LAB) at a 7 or a 10% NaCl concentration). Microbial, physicochemical and sensorial alterations monitored during the whole process, and several differences were observed between treatments. Results indicated that in all treatments, the dominant microflora were LAB. Yeasts also detected in noteworthy populations, especially in non-inoculated samples. However, LAB population was significantly higher in inoculated compared to non-inoculated samples. Microbial profiles identified by metagenomic approach showed meaningful differences between spontaneous and inoculated treatments. As a result, the profound dominance of starter culture had a severe effect on olives fermentation, resulting in lower pH and higher acidification, which was mainly caused by the higher levels of lactic acid produced. Furthermore, the elimination of *Enterobacteriaceae* was shortened, even at lower salt concentration. Although no effect observed concerning the quantitated organoleptic parameters such as color and texture, significantly higher levels in terms of antioxidant capacity were recorded in inoculated samples. At the same time, the degradation time of oleuropein was shortened, leading to the production of higher levels of hydroxytyrosol. Based on this evidence, the establishment of starter culture driven Picual olives fermentation is strongly recommended. It is crucial to mention that the inoculated treatment with reducing sodium content was highly appreciated by the sensory panel, enhancing the hypothesis that the production of Picual table olives at reduced NaCl levels is achievable.

## Introduction

Table olives are one of the most well-known fermented products, coming from the ancient time, being an essential element of the Mediterranean diet and culture (Benítez-Cabello et al., 2019). Nowadays, they constitute one of the most important fermented foods globally, with a production close to 2.7 million tons/year (International Olive Council [IOC], 2018). The main aims of olives processing are (i) the degradation of the secoiridoid oleuropein, which is responsible for the bitter flavor, making olives inedible products and (ii) the enhancement of organoleptic characteristics, making the final product acceptable by consumers (Zinno et al., 2017). Despite their significant role, olives fermentation remains empirical and thus an inconstant process. In recent years, process modernization, by the use of appropriate starter cultures, has been extremely supported, aiming to the stabilization of fermentation worldwide (Rodríguez-Gómez et al., 2017).

The directly brined olives, is among the most popular and established table olive commercial styles (Rodríguez-Gómez et al., 2017) and is mainly promoted by lactic acid bacteria (LAB) and yeasts. These microorganisms are usually coming from olive fruits and the processing environment (Corsetti et al., 2012). Major synergisms and interactions occur between dominant species until the end of fermentation, contributing to the improvement of sensorial attributes of the final product (Botta and Cocolin, 2012). Apart from the aforementioned, a plethora of undesired microbes may be involved, especially at the initial stage of the process, including many species belonging to *Enterobacteriaceae*, *Pseudomonas*, *Staphylococcus* and to a lesser extend *Clostridium* and molds (Bonatsou et al., 2017). All of them affect the course of fermentation and thus contribute to several characteristics of the product (flavor, texture, color, and safe consumption) (Heperkan, 2013). A critical point for the elimination of such microbial populations is the fast acidification of brines. It has been already reported the significant contribution of LAB for the brines acidification, due to the production of lactic acid, leading to the reduction pH and thus providing microbiological control and safety during fermentation (Papadelli et al., 2015; Sorrentino et al., 2016).

The microbial formation depends on olive cultivar, crop management, fermentation style, and technology processing type. This complex matrix can, in turn, contribute to the microbial consortia formation responsible for fermentation and influence the sensory and safety profile of the final product (Romeo, 2012; Perpetuini et al., 2020). Thus, both process standardization and an in-depth determination of the normal or abnormal microbial communities at different stages of fermentation are mandatory, in order not only to overpass such problems but also to identify potential biomarkers responsible for fermentation; thus learning how to manipulate fermentation conditions to improve the process control (Filippis et al., 2017). On that point, the use of classical molecular methods (culture-dependent techniques) do not offer a complete knowledge about the microbial consortia presented in food matrix ecosystem (Cocolin et al., 2013). Last decades, culture-independent techniques have attracted the attention of food scientific community. Nowadays, high-throughput sequencing (HTS) has revolutionized the field of food microbial ecology, as it has been established as a new tool for the quantitative investigation of the microbial communities’ structure (Medina et al., 2018). Several applications of next generation sequencing (NGS) technology can be already found in the frontiers of food microbiology (Ercolini et al., 2011; Bokulich et al., 2012) and the related crucial contribution regarding the enhancement of scientific knowledge, have been noted (Ercolini, 2013). NGS enables high-resolution studies of microbial composition (ranging from phylum to species level) in several food system categories, including food spoilage (Parlapani et al., 2018, 2019) and food fermentation (Ercolini et al., 2012; Cocolin et al., 2013; Ercolini, 2013). Therefore, this set of techniques has been recommended as an auspicious tool for the evaluation of the microbial diversity during fermentation of several products (Zinno et al., 2017). However, up to now, the monitoring of several dairies fermentations are by far the most explored products studied by such analyses (Fuka et al., 2013; Pasquale, 2014; O’Sullivan et al., 2015; Alessandria et al., 2016; De Filippis et al., 2016; Kamilari et al., 2020), while the investigation of other fermented products is more limited (Bokulich et al., 2014), including the monitoring of “microbial map” of olives fermentation (Randazzo et al., 2017).

From the beginning of the brining, a noteworthy amount of sodium (ranging from 8 to 13%) is added, aiming to provide both the safety of the process, as well as the improvement of some sensorial attributes of the processed product (Pino et al., 2018). Cypriot olive industry has established an amount of 10% salt content as a proper addition. However, according to the World Health Organization (World Health Organization [WHO], 2012), the daily proposed salt intake should not exceed 5 g. Consequently, one of the main aims of the industry is to align with this instruction. However, for the successful reduction of sodium levels, several parameters (cultivar, fruit composition, olives manipulation, processing type, use of appropriate starter culture) should be taken into consideration, before application at industrial scale (Bautista-Gallego et al., 2011).

Furthermore, despite that the fermentation profile of several olive cultivars has been already studied (Panagou et al., 2008; Bautista-Gallego et al., 2010; Tofalo et al., 2012; Bleve et al., 2014, 2015; Chranioti et al., 2018; Pino et al., 2018), limited information is available for the microbial and physicochemical changes during fermentation of Picual table olives (Tufariello et al., 2019). This cultivar is one of the primary produced olives in Cyprus, exhibiting high exports levels, as well.

Thus, the aims of this work were (a) to study the microbial communities and physicochemical changes of Picual (whole and cracked) table olives, during the fermentation process, (b) to evaluate the process’ evolution by adding a LAB starter culture, and (c) to study the effect of reducing NaCl concentration in combination with the starter culture, to produce a more secure and healthier final product.

## Materials and Methods

### Olives Samples and Fermentation Procedure

Olive fruits (*Olea europaea*) were supplied at the green stage of ripening, from a commercial orchard named “Novel Agro,” in Nicosia, Cyprus. After visual screening for the abortion of the damaged olives, fruits were thoroughly rinsed with tap water to reduce dust and other debris. Afterward, two types of olives (whole and cracked) subjected into three different fermentation treatments, in duplicate (biological replicate). Approximately 20 Kg of fruits were placed in plastic tanks of 25 L capacity filled with brine and citric acid solution, based on the local practices applied. Brine inoculation was applied immediately after brining (D0), according to manufacturer suggestion. Olives allowed to ferment for 120 days at controlled temperature (23 ± 2°C). The different types of fermentation are described in Table 1.

**TABLE 1:** Summary of the experimental design applied in the study.

**Olives**	**Description**	**Technology**
S1	Spontaneous fermentation (10% NaCl, citric acid 3.3%)	Cracked
S2	Spontaneous fermentation inoculated with starter culture *Lactobacillus plantarum* (final concentration 5 log cfu/ml) (Vege-Start 60′, Chr. Hansen A/S, Copenhagen, Denmark) (10% NaCl, citric acid 3.3%)	Cracked
S3	Spontaneous fermentation inoculated with starter culture *Lactobacillus plantarum* (final concentration 5 log cfu/ml) (Vege-Start 60′, Chr. Hansen A/S, Copenhagen, Denmark) (7% NaCl, citric acid 3.3%)	Cracked
S4	Spontaneous fermentation (10% NaCl, citric acid 3.3%)	Whole fruit
S5	Spontaneous fermentation inoculated with starter culture *Lactobacillus plantarum* (final concentration 5 log cfu/ml) (Vege-Start 60′, Chr. Hansen A/S, Copenhagen, Denmark) (10% NaCl, citric acid 3.3%)	Whole fruit
S6	Spontaneous fermentation inoculated with starter culture *Lactobacillus plantarum* (final concentration 5 log cfu/ml) (Vege-Start 60′, Chr. Hansen A/S, Copenhagen, Denmark) (7% NaCl, citric acid 3.3%)	Whole fruit

### Microbiological Analysis

Microbial changes were monitored at several time points (days 0, 8, 15, 22, 29, 45, 60, 90, 120), throughout the process. Brines were determined for their total viable count (TVC), LAB, Yeasts, *Enterobacteriaceae*, *coliforms*, and coagulase-negative staphylococci (CoNS) (Table 2), as described previously (Anagnostopoulos et al., 2020). Results expressed as log cfu/ml and analyses applied in triplicates.

**TABLE 2:** Microbiological media used for microbial enumeration.

**Growth media**	**Microorganisms**	**Method**	**Incubation conditions**
Plate Count Agar (PCA) (Merck, Darmstadt, Germany)	Total viable count	Spread plate	30°C/72 h
De Man-Rogosa-Sharpe agar (MRS) (Oxoid, Basingstoke, United Kingdom) + natamycin 0.1%	Lactic acid bacteria	Pour plate/Overlay	30°C/72 h
Sabouraud Agar (Oxoid, Basingstoke, United Kingdom)	Yeast and molds	Spread plate	25°C/5 days
Violet Red Bile Glycose Agar (VRBGA) (BD, Sparks, MD, United States)	Enterobacteriaceae	Pour plate/overlay	37°C/24 h
Violet Red Bile Lactose Agar (VRBLA) (Oxoid, Basingstoke, United Kingdom)	Coliforms	Pour plate/Overlay	30°C/24 h
Mannitol Salt Agar (MSA) (Oxoid, Basingstoke, United Kingdom)	CoNS	Spread plate	30°C/48 h

### 16S rRNA Meta-Barcoding Analysis

#### DNA Extraction, Quality Evaluation and Sequencing

Microbial genomic DNA from olives (day 0) and brine samples (days 60, 120) extracted as described by Medina et al. (2016), using the DNeasy^®^ PowerFood^®^ Microbial Kit (MoBio Laboratories Inc., Carlsbad, CA, United States). DNA concentration was measured on a Qubit 4.0 fluorimeter (Invitrogen, Carlsbad, CA, United States) using Qubit dsDNA HS Assay Kit (Invitrogen). The purity of DNA was evaluated by measuring the ratio of absorbance A260/280 nm and A260/230 nm using a spectrophotometer (NanoDrop Thermo Scientific, United States). Finally, the extracted DNA stored at −20°C until further use.

Bacterial diversity was assessed by sequencing the V3–V4 region of the 16S rRNA gene (expected size ∼460 bp) using the Illumina’s 16S Metagenomic Sequencing Library Preparation protocol (15,044,223 b). For the amplification of the V3–V4 region, two universal bacterial amplicon primers were used, including the amplicon PCR forward primer (TCGTCGGCAGCGTCAGATGTGTATAAGAGACAG) and amplicon PCR reverse primer (GTCTCGTGGGCTCGGA GATGTGTATAAGAGACAG) with the addition of the overhang adapter sequence. Nextera XT Index Kit (FC-131-2001, FC-131-2002) used for the multiplexing step. All PCR reactions were performed in a 96-well plate, incorporated in Thermocycler (Biorad), as described by Parlapani et al. (2019) PCR products were purified using NucleoMag^®^ NGS Bead Suspension (Macherey-Nagel, Düren, Germany), following by the second amplification using illumine sequencing indexes described above (FC-131-2001, FC-131-2002) (Nextera XT Index kit, Illumina Inc, San Diego, CA, United States). PCR reaction was applied as described in previous work (Parlapani et al., 2019). PCR products were purified using NucleoMag^®^ NGS Bead. The DNA concentration determined using Qubit 4.0 fluorimeter (Invitrogen, Carlsbad, CA, United States) and the quality was estimated using a bioanalyzer (Agilent 2200 TapeStation) (expected size ∼550 bp). Finally, at 4 pM of purified libraries were sequenced using the MiSeq^®^ reagent kit v2 (2 × 150 cycles) (Illumina, San Diego, CA, United States) in a MiSeq Illumina sequencing platform.

#### Bioinformatics and Data Analysis

The analysis was applied using the 16S Metagenomics app (version 1.1.0) from the Illumina BaseSpace platform and sequences with Phred < Q20 were removed. Operational taxonomic units (OTUs) were created, using Ribosomal Database Project Classifier (Wang et al., 2007) against the Illumina-curated version of GreenGenes reference taxonomy database (DeSantis et al., 2006), as described previously (Yuan et al., 2018). The classified OTUs were defined at ≥97% of sequence homology and converted to percentages (relative abundances), to determine the representation of each microbe among treatments. Alpha diversity indexes were calculated using the EstimateS version 9.1.0^1^, by different metrics (Chao, Shannon, Simpson) after performing a rarefaction analysis. Rarefied OTU to 54,488 sequences (lowest number of reads obtained) were used to obtain these indices. Finally, all sequences were deposited to the National Centre for Biotechnology Information (NCBI) in Sequence Read Archive (SRA) under the BioProject PRJNA600153.

### Physicochemical Analysis

The changes of pH were measured using a pH meter (Hanna Instruments), while titratable acidity (TA) was estimated by titration of brines with 0.1 mol L^–1^ NaOH up to pH 8.3. Results expressed as a percentage of lactic acid equivalent (w/v). Electrical conductivity was measured using a conductivity meter (Mettler Toledo), while water potential was monitored using a potentiometer dewpoint (WP4C), following the manufacturer’s instructions. All analyses were performed in triplicates.

The monitoring of organic acids (lactic, succinic, tartaric, acetic, citric, and malic) sugars (glucose, sucrose, and fructose), and alcohols (ethanol and glycerol) alternations were applied using high pressure liquid chromatography (HPLC) (Waters 1525), as described previously (Bonatsou et al., 2018).

The extraction of the phenolic compounds was achieved following the protocol described by Tataridou and Kotzekidou (2015). The extracts were stored at −20°C until further use. The determination of total polyphenols was applied as described by Uylaşer (2015), using the Folin-Ciocalteu (F-C) reagent. The reacted samples were measured spectrophotometrically (765 nm). Results were expressed as mg/g of gallic acid equivalent (GAE). Continuously, the antioxidant activity of phenolic extracts was estimated spectrophotometrically (517 nm) according to D’Antuono et al. (2018), using the free radical 2,2-diphenyl-1-picrylhydrazyl (DPPH) (Sigma-Aldrich, Taufkirchen, Germany). The results were expressed as mg/g of Trolox equivalent antioxidant capacity (TEAC) fresh weight, using the standard curve of Trolox. Analyses performed three times.

Finally, the changes of the main phenolic compounds (oleuropein and hydroxytyrosol) determined chromatographically using HPLC (Waters 1525), as described by Anagnostopoulos et al. (2020). Results were expressed as means (mg/g or mg/ml) and standard deviations of three replicates.

### Color and Texture Profile

Color alternations were monitored using a CR200 Chroma Meter (Minolta camera Co., Ltd.), as described previously (Anagnostopoulos et al., 2020). The analysis performed in ca. 10 different olives from each treatment.

Texture evaluation was estimated using a texture analyzer (Stable Micro Systems TA.XT*plus*C, Vienna Court, Lammas Road, Godalming, Surrey GU7 1YL, United Kingdom) following the method of Fadda et al. (2014). The analysis applied to at least ten random olives.

### Sensory Analysis

All treatments were evaluated at the end of the process (120 days) by 13 panelist members according to Anagnostopoulos et al. (2020).

### Statistical Analysis

Microbial, physicochemical and sensorial data were analyzed by analysis of variance (one-way ANOVA), using the SPSS 20 software (StatSoft Inc., Tulsa, OK, United States), to underline any significant difference among treatments. The test used was the Least Significant Difference (LSD) at the significance level of 0.05. Furthermore, principal components analysis (PCA) was performed, using varimax rotation, to determine potential distances between the different treatments, during the fermentation process. Factors exhibiting eigenvalues greater than 1.0 were retained, for the selection of the optimum number of principal components (PCs). Afterward, scores were projected into plots by the first two components.

## Results

### Microbiological Analysis

**FIGURE 1:**
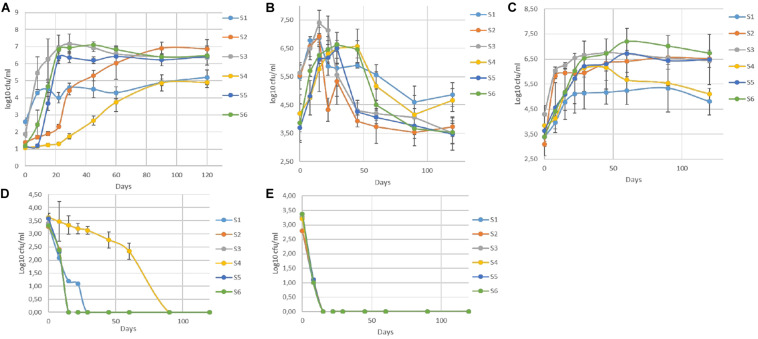
Evolution of microbial changes throughout the fermentation of different treatments (S1–S6) LAB **(A)**, yeasts **(B)**, TVC **(C)**, *Enterobacteriaceae* **(D),** and *coliforms* **(E)**. Results were expressed as log10 cfu/ml of three replicates ± standard deviation.

The population dynamics of TVC, LAB, yeasts, (CoNS), *coliforms*, and *Enterobacteriaceae* during fermentation are presented in Figure 1. In general, LAB and yeasts were the principal members of the microflora in all treatments. Differences were recorded mainly between non-inoculated and inoculated samples, regardless of olive’s technology (cracked or whole). Results indicated that LAB predominated in all treatments, while the yeast population was noteworthy, especially in non-inoculated treatments (S1, S4). On the other hand, (CoNS)were undetectable at all time points, while *Enterobacteriaceae* and *coliforms* decreased after the first days. They were detected at an average of 3.5 log cfu/ml at the beginning of the process, but they decreased rapidly and could not be detected after 22 days of fermentation, with an exception in S4, where they were detectable until 60th day. It is worth noted the faster elimination of *Enterobacteriaceae* in all inoculated treatments (15 days) compare to the other non-inoculated sample (S1). In the yeast population, an initial increase phase was observed in all cases, reaching the maximum level of approximately 6.5 log cfu/ml at circa 15 days. Following, a decrease was observed in all treatments, until day 60 and then remained steady. However, the decrease in inoculated olives was significantly higher, with an average reduction of about 2.5 log (from 6.5 log cfu/ml at day 15 to 4 log cfu/ml at day 60) (Figure 1B). At the end of fermentation, the population in non-inoculated samples (S1, S4), which had no significant differences with each other, exhibited an average value of 4.7 log cfu/ml, while in the other treatments was more limited. Regarding LAB, a steady trend in samples inoculated with the starter culture (S2, S3, S5, S6), was recorded, where an initial population of about 2 log cfu/ml (sampled before inoculation) followed by a significant increase of approximately 4 log at day 60 and after that remained steady until the end of fermentation, reaching an average population of 6.6 log cfu/ml.

Although there were no differences at the end of fermentation, the slower increase of LAB population in S2, the first 45 days of the process is notable. Oppositely, non-inoculated samples exhibited significantly lower LAB populations, reaching a value of 5 log cfu/ml at the 120th day. However, as in inoculated treatments, LAB were the predominant species in S1 and S4 treatments, at the end of the process. Similar behavior observed for TVC, in which the highest concentration value in all inoculated samples was reached after 45 days of fermentation. The highest value (7 log cfu/ml) was detected in sample S6, while the lower populations recorded for both non-inoculated treatments (S1, S4) at the end of the process (4.9 log cfu/ml).

### Metagenomic Analysis

#### Illumina MiSeq Data Analysis

Overall, after the removal of poor quality sequences, a total of 4,170,737 sequences (an average of 231,707 sequences per sample per day) were obtained for data analysis. Table 3 also shows the total number of OTUs found in the different samples throughout days and their alpha-diversity indexes. In general, higher biodiversity indicated for treatments S4 and S5, which showed the highest values for Simpson and Shannon indexes. It is interesting that among treatments, the highest biodiversity observed at the end of the process. The total number of OTUs assigned ranged from 85 to 272, with an average of 178 detected OTUs per sample. A mean value of ca. 231,707 reads were analyzed, with the highest value recorded (621,655) for the S6 sample at the 120th day and the lowest (54,488) for the S1 sample, at the beginning of fermentation. Regarding chao1 index, our results indicated that most of the bacterial diversity is present in the samples, since this index was higher compare to the observed OTUs, in all treatments.

**TABLE 3:** Number of sequences and OTUs assigned, diversity indexes, and estimated sample coverage for 16S (bacteria) amplicons according to different treatments (S1–S6 treatments), D: days.

**Sample**	**Number of reads**	**Number of OTUs**	**Simpson**	**Shannon**	**Chao1**
S1_D0	54,488	272	1.71	1.13	436.31
S1_D60	71,360	163	1.71	1.12	465.92
S1_D120	102,972	133	1.71	1.12	457.36
S2_D0	117,071	260	1.71	1.12	451.7
S2_D60	119,799	254	1.72	1.13	442.85
S2_D120	130,925	210	1.73	1.14	433.44
S3_D0	137,191	268	1.73	1.13	425.13
S3_D60	151,817	91	1.73	1.13	412.11
S3_D120	157,961	97	1.74	1.13	398.4
S4_D0	168,025	285	2.08	1.11	266.37
S4_D60	212,689	85	2.31	1.11	220.03
S4_D120	241,137	215	3.64	1.38	365.81
S5_D0	271,235	252	1.81	1.12	340.9
S5_D60	331,000	235	1.9	1.14	319.18
S5_D120	363,295	129	2.11	1.15	294.78
S6_D0	382,718	258	1.75	1.13	387.35
S6_D60	535,399	175	1.76	1.12	370.7
S6_D120	621,655	90	1.75	1.11	160.02

#### Bacterial Diversity

**FIGURE 2:**
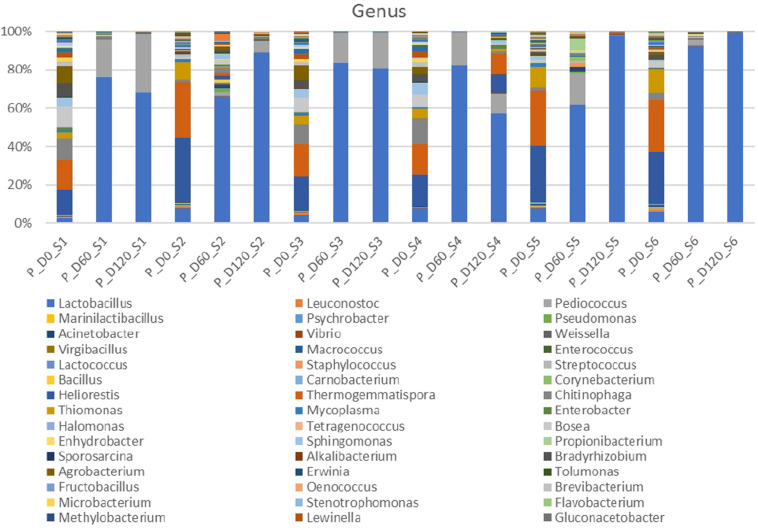
Relative abundance of bacterial genera in the different treatments assayed (S1–S6), obtained through metagenetic analysis of 16S RNA gene at the initial (D0), middle (D60), and the end (D120) of the fermentation process.

Metagenomic analysis of 16s rRNA indicated that three main bacterial phyla detected in high relative abundance (*Firmicutes, Proteobacteria, Bacteroides*) and another four to a lesser extend (*Actinobacteria, Chloroflexi, Tenericutes, Verrucomicrobia*) (Supplementary Figure 1). *Proteobacteria* and *Bacteroidetes* were the most abundant phyla at the beginning of the process, following by *Firmicutes*. However, *Firmicutes* predomination was profound at the middle and the end of fermentation. Figure 2 depicts the bacterial genera detected in all treatments. At the beginning of the process, genera such as *Thermogemmatispora, Chitinophaga, Agrobacterium, Thiomonas, Bosea, Bradyrhizobium, Heliorestis* and to a lesser extend *Lactobacillus*, were the most frequently detected in all samples On the contrary, mainly *Lactobacillus* and to a lesser extend *Pediococcus* became the most abundant genera at days 60 and 120 in all treatments, while the former had higher relative abundance in inoculated treatments. It is crucial to mention that the relative abundance of *Pediococcus* was higher in cracked samples at the end of the process. Finally, *Thermogemmatispora, Chitinophaga, Agrobacterium*, and *Heliorestis* were also found at noteworthy abundances in non-inoculated treatment S4 at the end of fermentation.

**FIGURE 3:**
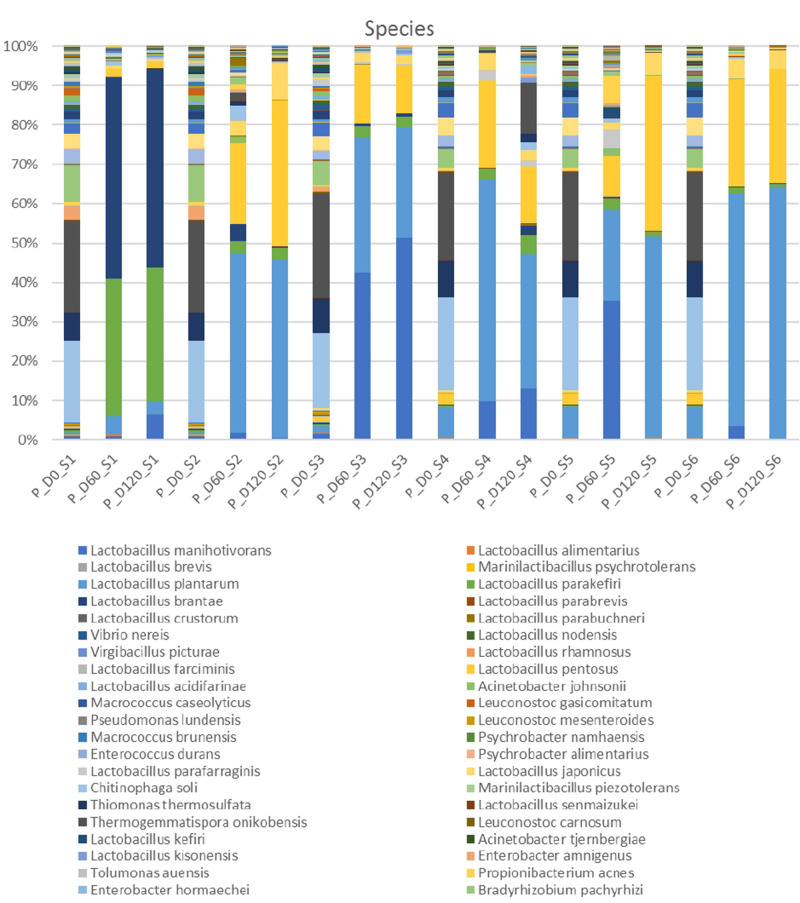
Relative abundance of bacterial species in the different treatments assayed (S1–S6), obtained through metagenetic analysis of 16S RNA gene at the initial (D0), middle (D60), and the end (D120) of the fermentation process.

In the species level, non-inoculated and inoculated treatments recorded differences in their microbiome evolution (Figure 3). NaCl reduction, also had a slight impact on the microbial communities composition. In day 0, the most abundant species were *Thermogemmatispora onikobensis* and *Chitinophaga soli*, followed by *Thiomonas thermosulfata* and *Bradyrhizobium pachyrhizi*, while *Lewinella lutea*, *Brevibacterium casei*, as well as *Lactobacillus plantarum* group were more limited. However, in the middle of fermentation (day 60), several *Lactobacillus* spp. and mainly *L. plantarum* group and *Lactobacillus manihotivorans* became the predominant flora in inoculated treatments. These species were found to dominate at the 120th day as well, while their relative abundances were equal to day 60. On the contrary, different microbiome profile was observed in S1 treatment, where *Lactobacillus brantae* and *Lactobacillus parakefiri* were by far the most dominant species, while *L. plantarum* group and *L. manihotivorans* were detected at low levels. As for sample S4, which had the highest biodiversity at the end of the process, it exhibited a quite unique microbial profile. Although *L. plantarum* group was the most abundant species, followed by *L. manihotivorans*, the presence of *T. onikobensis* and *T. thermosulfata* is noteworthy at the end of the process, while *Lactobacillus japonicus*, *L. brantae, Lactobacillus parafaraginis*, and *L. parakefiri* were also detected, at lower levels. Notably, species such as *Enterobacter hormaechei*, *Agrobacterium viscosum*, and *Mycoplasma insons* were only detected in this treatment, at the end of the process.

### Physicochemical Analysis

**FIGURE 4:**
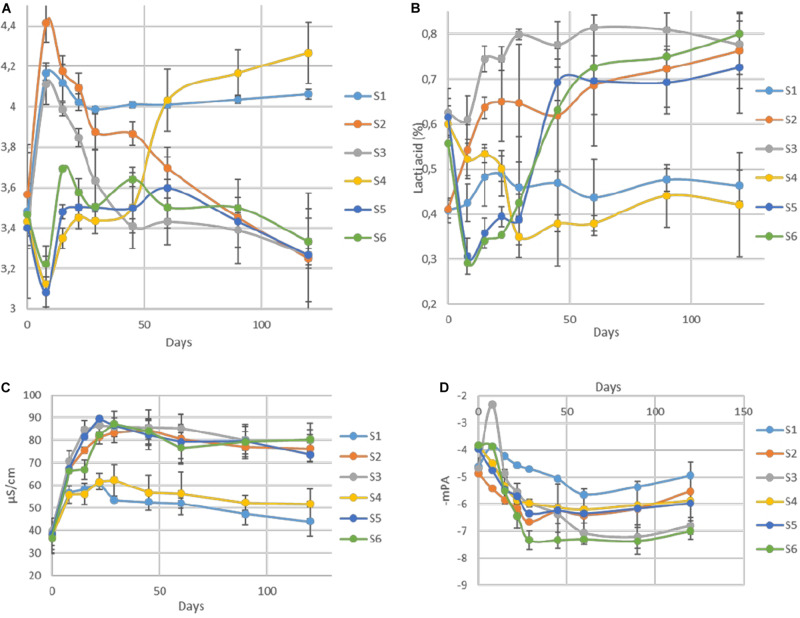
Changes in pH **(A)**, titratable acidity **(B)**, electrical conductivity **(C)**, and water potential **(D)** throughout the fermentation of different treatments (S1–S6). Results are expressed as means and standard deviations of three replicates.

The changes of pH in the brines during fermentation are presented in Figure 4A. During the first days of the process (until day 29), a differentiation of acidic conditions based on olive’s technology (cracked and whole fruit) was observed. Afterward, samples were grouped based on spontaneous and inoculated treatments. More specifically, the initial pH values (day 0) in all treatments were very low (3.4) due to the use of citric acid at the beginning of fermentation. Then, an increase of approximately 0.4–1 unit until day 8 was observed in cracked olives (S1, S2, S3), while a slight decrease of about 0.3 units was recorded in whole fruits (S4, S5, S6). After that point, a major decrease of pH was recorded in inoculated treatments, reaching an average value of 3.4 at day 90, which remained unchanged until the end. On the other hand, between non-inoculated treatments, no significant differences were observed between 60 and 90 days, reaching significantly higher pH (4). However, at the end of the process, the pH of S4 was raised further (4.2). Concerning TA (Figure 4B), results indicated that after the first period (ca. 29 days), where a dispersal between treatments was observed, there was a clear differentiation between non-inoculated and inoculated treatments, in agreement with pH findings. At the end of the process, the average acidity of non-inoculated and inoculated treatments was 0.45 and 0.77% lactic acid, respectively. Moreover, significant differences observed in electrical conductivity (Figure 4C) between non-inoculated and inoculated samples, which recorded from the first days of fermentation. Inoculated samples exhibited significantly higher values during the whole process, reaching the highest rates at 29th day (87 μs/cm), while values in non-inoculated treatments were more limited, reaching an average value of 45 μs/cm at the end of the process. Finally, water potential (Figure 4D) showed significant differences between whole and cracked treatments at the initial stage of the process, while after 60 days of fermentation, treatments were grouped according to sodium content. Specifically, treatments S3 and S6 exhibited significantly lower values than the others.

**FIGURE 5:**
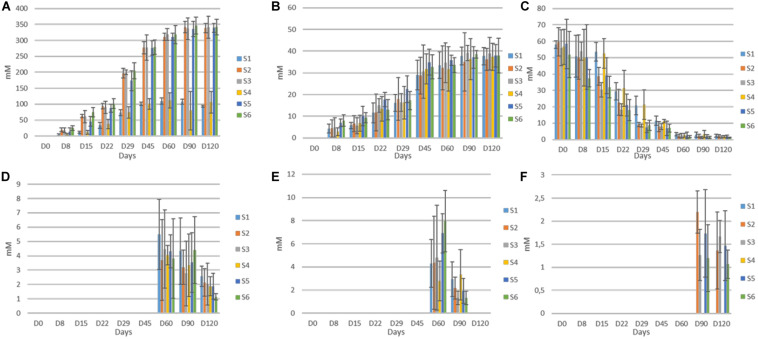
Changes in the concentration (mM) of organic acids (lactic, **A**; acetic, **B**; citric, **C**; malic, **D**; tartaric, **E**; and succinic, **F**) throughout the fermentation of different treatments (S1–S6). Data points are expressed as means and standard deviations of three replicates.

The evolution of organic acids in the brines during fermentation is presented in Figure 5. Overall, significant differences between un-started and inoculated treatments observed, as detected by HPLC analysis. More specific, citric was the main acid at the initial stage in all treatments, due to its use at the beginning of the fermentation. After approximately 22 days, lactic and to a lesser extend acetic became the main acids in all treatments, while an almost total depletion of citric was observed, after 60 days of fermentation. Although lactic acid was the dominant element in all treatments, its levels were significantly higher in inoculated treatments, throughout the process, while no differences recorded for acetic acid. Furthermore, mallic and tartaric acids were also found in traces after 60 days, while the latter was totally disappeared at the end of the process. No differences recorded between treatments regarding those acids. Finally, succinic acid was also detected in traces, only in the inoculated treatments, after 90 days of fermentation. No distributions were recorded thereafter.

**FIGURE 6:**
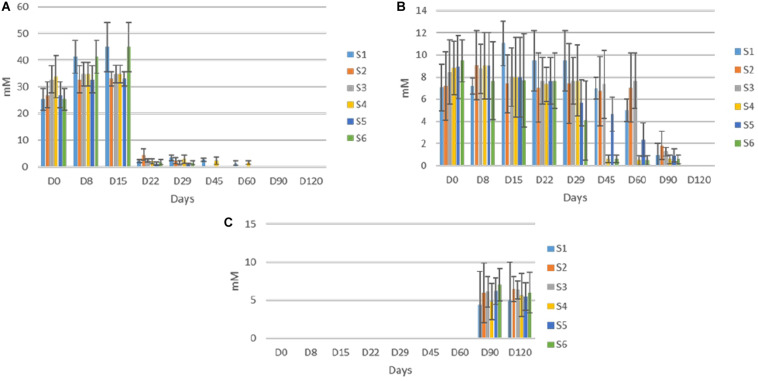
Changes in the concentration (mM) of soluble sugars (glucose, **A**; fructose, **B**); and ethanol, **C** in the brines throughout the fermentation of different treatments (S1–S6). Data points are expressed as means and standard deviations in triplicate.

Concerning soluble sugars, glucose and fructose were the major sugars in the brines detected by HPLC (Figure 6). The content of both rapidly increased at the early stage of fermentation and reached a maximum after 15 days of fermentation. Then, a significant decrease recorded, while neither glucose nor fructose were detectable at the end of the process. The glucose depletion ascended faster in inoculated treatments, as it was undetectable after 45 days of fermentation. A similar pattern observed for fructose with two differences. Firstly, no differences observed between treatments regarding fructose consumption. Secondly, fructose was detectable for 90 days of the process. Finally, sucrose was never detected in any treatment, indicating the absence of this element in olive fruit. Regarding ethanol and glycerol, the former was detected in all treatments in low amounts (no significant differences) after 90 days of the process, while the latter observed in traces only in S1 treatment at the 120th day (data not shown).

**FIGURE 7:**
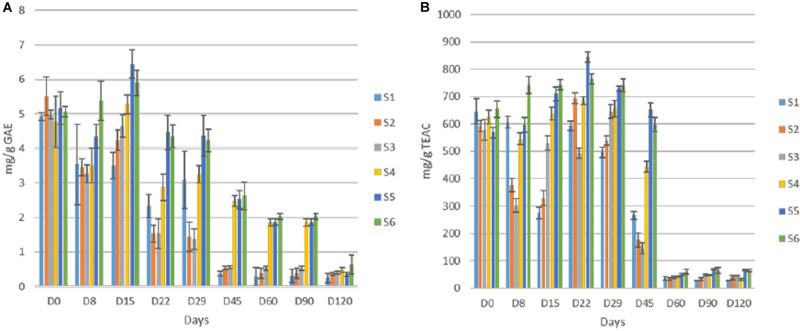
Total phenolic content **(A)** and antioxidant capacity of olives **(B)** during fermentation of different treatments (S1–S6). Results were expressed as means and standard deviations of three replicates, equivalent of mg/g.

Total polyphenols detected in olive fruits showed quite differences among cracked and whole olives, while within each group similarities on TP content was achieved (Figure 7A). The initial value was close to 5 mg/g GAE in all treatments, but the reduction was faster in cracked olives (S1, S2, S3), reaching an average value of 0.5 mg/g at 45th day, while the TP of S4, S5, S6 was significant higher (ca. 2.45 mg/g) at this time point. However, at the end of the process, no significant differences were observed in any of the treatments. Additionally, the loss in phenolic compounds resulted in a remarkable loss of antioxidant capacity in olive fruits, as well (Figure 7B). However, in line with TP reduction, the antioxidant capacity reduction was significantly faster in cracked olives, while differences between cracked and whole olives observed until 45th day. After 60 days of fermentation, a major decrease (ca. 90%) in whole fruits indicated. It is crucial to mention that at the end of the process, significantly higher values were recorded in inoculated treatments, while S1 exhibited the lowest antioxidant capacity (27.4 mg/g).

**FIGURE 8:**
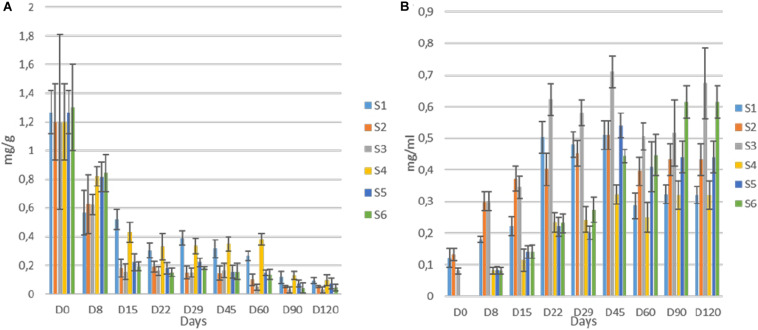
Evolution oleuropein **(A)** and hydroxytyrosol **(B)** during fermentation of different treatments (S1–S6). Results were expressed as means (mg/g or mg/ml) and standard deviations of three replicates.

Regarding oleuropein monitoring, significant degradation was observed in all treatments as fermentation time passed. The degradation was significantly faster in inoculated treatments (Figure 8A) though. More specifically, the loss of oleuropein in S2, S3, S5, and S6 was close to 92% at day 45, while the equal loss in whole fruits was quite lower (ca. 70%) at the same time point. No differences observed within these two groups. Moreover, at the end of the process, oleuropein was detected in traces, while still recorded lower concertation in inoculated samples. Regarding hydroxytyrosol, an exponential increase for all treatments until the end of the process observed, where higher levels recorded in samples S3 and S6 (Figure 8B). However, during fermentation, the production of hydroxytyrosol was higher in cracked olives, exhibiting higher levels than in whole fruits until day 29. After that point, it seems that the production of hydroxytyrosol was higher in inoculated treatments, regardless of fruit technology. At the end of the process, inoculated treatments with 7% NaCl content (S3, S6) exhibited the highest values (ca. 0.64 mg/ml), followed by the other inoculated samples (S2, S5) while the lowest concentrations recorded for non-inoculated olives, with an average value close to 0.32 mg/ml.

### Firmness and Color Evolution of Olives

Regarding texture (Table 4), a continuous decreasing tendency was observed, as time passes, until ca. 29 days. After, no changes were recorded in any of treatments, until the end of the process. Samples clearly grouped based on technology, as the firmness of cracked olives were significantly lower during the whole fermentation. No differences observed within groups.

**TABLE 4:** Evolution of texture of olive fruits during fermentation of different treatments (S1–S6).

**DAY**	**S1**	**S2**	**S3**	**S4**	**S5**	**S6**
0	19 ± 2	19.66 ± 5.13	20 ± 2.64	49.46 ± 2.9	50.03 ± 1.77	53.5 ± 3.66
8	18.33 ± 2.08	18.33 ± 1.15	19.33 ± 3.05	32.26 ± 4.11	33.62 ± 4.13	32.96 ± 2
15	12.66 ± 1.52	14 ± 2.64	14.66 ± 2.51	20.86 ± 4.55	24.58 ± 3.18	21.83 ± 0.83
22	11.33 ± 1.52	11.66 ± 1.52	12 ± 2.64	18.46 ± 3.13	19.6 ± 1.29	18 ± 3.6
29	7.33 ± 2.08	7 ± 1	8.3 ± 0.3	17.16 ± 3.32	18.9 ± 1.13	18.36 ± 0.56
45	6.23 ± 0.2	5.96 ± 0.55	6.43 ± 0.8	17.3 ± 1.57	18.23 ± 1.68	17.37 ± 0.54
60	6.16 ± 0.15	5.91 ± 0.72	6.53 ± 0.64	17.8 ± 2.3	18.2 ± 1.9	17.26 ± 0.64
90	6.06 ± 0.15	5.6 ± 0.78	6.4 ± 0.65	17.03 ± 1.95	17.6 ± 1.47	17.25 ± 0.64
120	6 ± 0.09	5.53 ± 0.75	5.93 ± 0.55	16.66 ± 1.52	17.54 ± 1.44	17.1 ± 0.78

*Data points are expressed as means (N) and standard deviations of at least 10 random measurements.*

Regarding color analysis, all parameters are presented in Table 5. In general, no significant differences observed for most of color parameters. However, it is crucial to mention that the loss in greenness was higher in samples with reduced NaCl content (S3, S6). Nevertheless, it is notable the similar values of all treatments at the end of the fermentation process, with no significant differences.

**TABLE 5:** Evolution of color parameters (a*, b*, L*, h*, and C*) of different treatments (S1–S6) during fermentation of Picual table olives.

**Sample**	**S1**	**S2**	**S3**	**S4**	**S5**	**S6**
**DAY**						
a*						
0	−13.18 ± 3.30	−14.44 ± 6.14	−15.56 ± 1.90	−19.22 ± 1.93	−18.87 ± 0.61	−19.54 ± 1.33
8	−6.85 ± 1.34	−6.23 ± 1.65	−6.49 ± 3.20	−17.61 ± 3.71	−16.04 ± 2.77	−11.20 ± 2.68
15	−3.47 ± 0.64	−5.78 ± 0.09	−6.49 ± 3.20	−6.49 ± 3.20	−14.38 ± 2.71	−11.20 ± 4.15
22	−0.87 ± 0.12	−5.78 ± 0.09	−2.78 ± 0.42	−3.34 ± 4.08	−4.13 ± 1.78	−2.23 ± 2.77
29	−0.33 ± 0.53	−3.15 ± 2.44	−3.08 ± 0.34	−2.37 ± 1.39	−3.24 ± 0.75	−1.08 ± 1.11
45	−0.82 ± 1.24	−1.98 ± 2.32	−1.43 ± 1.09	0.03 ± 0.77	−1.31 ± 3.07	0.35 ± 2.72
60	−0.45 ± 0.72	−2.18 ± 1.34	−1.31 ± 0.89	0.22 ± 1.18	1.03 ± 1.40	1.24 ± 3.51
90	0.37 ± 0.55	−2.18 ± 1.34	1.47 ± 0.45	0.22 ± 1.18	−1.92 ± 2.86	1.24 ± 3.51
120	0.88 ± 0.55	−2.30 ± 1.72	1.73 ± 0.22	0.22 ± 1.18	−1.92 ± 2.86	1.57 ± 2.42
b*						
0	38.72 ± 2.94	37.41 ± 4.68	38.70 ± 1.62	41.14 ± 0.34	36.85 ± 1.95	37.21 ± 2.25
8	34.34 ± 2.72	32.72 ± 6.14	35.39 ± 3.33	39.06 ± 2.31	35.53 ± 3.67	37.43 ± 2.86
15	31.53 ± 2.17	32.62 ± 6.11	34.63 ± 2.04	39.06 ± 2.31	35.53 ± 3.67	37.43 ± 2.86
22	29.16 ± 0.56	34.07 ± 0.48	31.68 ± 1.29	32.07 ± 7.41	33.75 ± 1.21	35.02 ± 1.72
29	29.13 ± 3.94	31.64 ± 2.72	32.61 ± 3.89	28.72 ± 3.91	31.76 ± 2.16	33.34 ± 0.26
45	28.69 ± 2.10	33.04 ± 5.27	32.53 ± 1.44	29.22 ± 5.91	29.47 ± 5.64	34.02 ± 2.84
60	31.70 ± 2.36	32.97 ± 4.18	32.64 ± 3.66	29.80 ± 4.66	30.71 ± 0.37	34.85 ± 1.86
90	32.11 ± 4.92	31.95 ± 5.71	31.20 ± 4.66	29.27 ± 4.26	30.87 ± 3.56	34.15 ± 3.30
120	32.11 ± 4.92	33.13 ± 6.32	31.20 ± 4.66	29.27 ± 4.26	30.87 ± 3.56	34.15 ± 3.30
L*						
0	60.01 ± 3.55	57.56 ± 5.25	57.84 ± 2.81	59.25 ± 0.95	54.06 ± 2.71	56.44 ± 3.75
8	57.15 ± 5.12	53.85 ± 7.55	55.50 ± 6.08	56.97 ± 1.32	55.61 ± 5.52	56.68 ± 5.84
15	53.17 ± 2.60	54.89 ± 7.33	55.30 ± 6.23	55.13 ± 4.72	52.86 ± 0.81	57.08 ± 5.50
22	52.23 ± 1.70	54.35 ± 0.48	52.13 ± 0.08	54.75 ± 4.36	54.08 ± 0.67	53.97 ± 0.39
29	53.33 ± 2.18	52.14 ± 0.83	54.59 ± 2.52	53.13 ± 3.77	53.23 ± 1.94	54.32 ± 0.69
45	51.78 ± 2.99	54.74 ± 2.16	53.56 ± 1.48	52.07 ± 3.11	53.06 ± 6.78	57.53 ± 6.24
60	54.15 ± 4.24	52.65 ± 5.39	55.79 ± 4.38	52.15 ± 2.38	50.40 ± 3.06	58.15 ± 5.87
90	54.15 ± 4.24	52.65 ± 5.39	50.86 ± 0.66	52.15 ± 2.38	50.40 ± 3.06	58.15 ± 5.87
120	56.50 ± 5.15	55.42 ± 6.83	50.86 ± 0.66	53.76 ± 5.57	51.04 ± 4.96	55.76 ± 2.11
C*						
0	40.96 ± 3.65	40.26 ± 6.39	41.74 ± 1.46	45.42 ± 1.03	41.40 ± 1.98	41.65 ± 1.66
8	35.02 ± 2.81	33.32 ± 6.24	36.04 ± 3.89	42.90 ± 3.53	39.01 ± 4.28	39.71 ± 3.61
15	33.59 ± 4.78	33.32 ± 6.24	35.83 ± 4.13	40.94 ± 2.86	38.14 ± 5.38	39.25 ± 4.12
22	29.17 ± 0.56	34.84 ± 0.49	31.80 ± 1.32	34.65 ± 8.76	36.20 ± 3.29	36.24 ± 0.77
29	30.84 ± 1.20	30.14 ± 1.06	33.38 ± 3.15	31.06 ± 1.33	32.46 ± 1.80	33.09 ± 0.75
45	29.24 ± 2.86	31.61 ± 6.80	32.17 ± 1.61	30.93 ± 4.42	30.13 ± 5.88	33.70 ± 3.07
60	31.58 ± 2.58	32.16 ± 5.58	32.99 ± 3.27	31.52 ± 1.82	28.38 ± 4.24	34.49 ± 2.74
90	31.58 ± 2.58	35.06 ± 2.54	32.99 ± 3.27	31.52 ± 1.82	28.38 ± 4.24	34.49 ± 2.74
120	32.89 ± 3.64	34.49 ± 4.30	29.72 ± 2.11	30.97 ± 1.57	28.68 ± 5.71	34.76 ± 3.13
h*						
0	108.62 ± 3.59	110.44 ± 6.51	111.92 ± 2.78	112.41 ± 3.33	117.13 ± 0.68	116.67 ± 2.77
8	101.25 ± 1.91	100.79 ± 2.05	100.11 ± 3.91	114.08 ± 3.54	114.33 ± 2.73	109.30 ± 2.21
15	93.78 ± 4.23	98.91 ± 1.82	98.96 ± 2.69	96.89 ± 2.45	98.51 ± 2.19	101.74 ± 1.41
22	91.71 ± 0.20	98.43 ± 2.10	95.79 ± 1.73	92.35 ± 5.74	97.78 ± 2.32	98.88 ± 3.66
29	89.46 ± 1.43	94.67 ± 3.87	95.69 ± 1.43	93.16 ± 2.60	97.12 ± 2.21	93.56 ± 2.17
45	91.30 ± 2.64	94.24 ± 2.44	91.93 ± 0.93	88.33 ± 2.14	92.51 ± 2.80	91.81 ± 2.71
60	89.18 ± 3.01	93.65 ± 1.77	92.97 ± 1.49	89.52 ± 3.38	90.78 ± 3.30	93.54 ± 5.03
90	90.05 ± 4.45	94.41 ± 1.24	90.74 ± 3.01	87.55 ± 3.16	96.07 ± 1.10	93.63 ± 2.10
120	88.56 ± 2.22	92.61 ± 1.28	91.33 ± 1.53	88.81 ± 6.06	93.86 ± 3.00	92.87 ± 3.01

*Data points are expressed as means and standard deviations of 10 random measurements.*

### Sensory Evaluation

**FIGURE 9:**
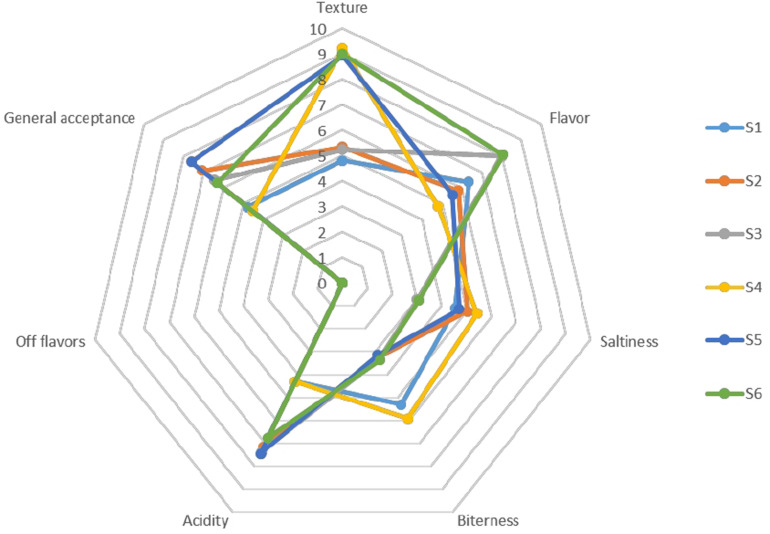
Sensory profiles of different treatments of Picual table olives after 120 days of fermentation.

The organoleptic profile of olives after 120 days of fermentation is presented in Figure 9. In general, the products characterized by acceptance taste and aroma, low bitterness and satisfied crispness, while no-off flavors noted, as inferred by the low scores of the taste panel for this organoleptic perception in all treatments. Generally, regarding the gustatory sensations, differences among treatments were detected on bitterness descriptor, in which non-inoculated treatments had a higher score from the inoculated ones. Regarding saltiness score, lower values were scored in S3 and S6 samples, while the texture of whole fruits was significantly higher than in cracked. However, inoculated treatments had the highest amount of acidity, receiving the highest score for the overall acceptability descriptor, except for S6 sample, which showed an average score between inoculated and non-inoculated scores. Notably that olives with lower NaCl content exhibited higher score regarding flavors. As a result, it seems that the main parameters affected the preference of the panel were flavor, acidity and bitterness to a letter extend.

### Multivariate Analysis

**FIGURE 10:**
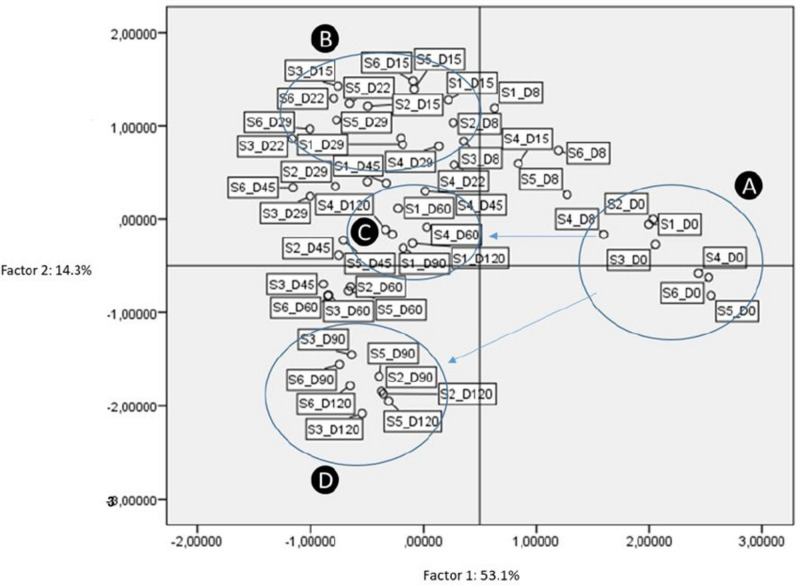
Plot of scores and loadings between treatments formed by the first two principal components from the PCA analysis. Labeling of data points indicate the processing treatment of olives (S1–S6), and fermentation time (D: days). A, B, C, D are the four main groups resulted according to analysis.

According to PCA, four factors retained (eigenvalue more than 1). These factors explained 83.1% of distribution, while the first two factors had a sum of 67.4% of the total variance (Figure 10). According to analysis, treatments grouped in four main clusters, based on inoculation. The latter is obvious, especially in the last days of fermentation time (Groups C and D, respectively). As clearly observed, non-inoculated treatments were separated from the others from 45th day and thereafter. Thus, it was evident that inoculation was the main factor responsible for the discrimination of treatments during the process. It is crucial to mention that the reduction of NaCl concentration (S3 and S6) did not affect the groups’ distribution.

## Discussion

Table olives are fermented fruits with a great impact on the Mediterranean diet. Due to their high nutritional value and their antioxidant capacity, they considered to be important functional food. Despite their significant importance, the fermentation is still an empirical method, coming from ancient times. It is driven spontaneously by the indigenous microflora and is not a stable process, usually depending on year, technology and processing. On that point, a current challenge in the processing technology of table olives is the establishment of using starter cultures, able to standardize, accelerate and safely drive the fermentation process (Pino et al., 2019). In parallel, a second challenge for the industry is the reduction of NaCl content, which is in high levels, ranging from 8 to 13% among producer countries, while in Cyprus, the average use of NaCl content is 10%, aiming to reduce undesirable spoilage and pathogenic microorganisms, ensuring the microbiological safety and quality of the final product. However, nowadays, based on both consumer’s demand and regulatory authorities, the reduction of sodium intake is a must (Zinno et al., 2017). The use of alternative salts is among the main widely applied strategies to reduce sodium content in table olives (Bautista-Gallego et al., 2015). Limited studies have tried to produce table olives with low NaCl content, in combination with the use of starter culture, without adding any salt replacement (De Bellis et al., 2010; Pino et al., 2018, 2019). Moreover, to our knowledge, there is limited awareness in those aspects concerning fermentation of Picual table olives. Only Tufariello et al. (2019) investigated the fermentation of Picual with the use of yeast starters. However, the study applied in high NaCl content (11%). Based on those evidence, the present study is the first to investigate the application of LAB starter and the reduction of sodium content for Picual table olive production, to evaluate if this strategy can potentially ensure and favor the quality and safety of fermentation process and the final product.

Overall, according to our results, the main differences observed across treatments were mainly related to the inoculation with LAB starter, while slight effect recorded between different salt content (7 and 10%) and technology of olives (cracked or whole fruit). This was also confirmed by PCA, where treatments were clearly separated based on inoculation. These findings indicate that inoculation could drive the fermentation, regardless of salt content and other aspects, to more controlled conditions, resulting in a stable and reproducible final product. Based on this hypothesis, we strongly support that the reduction of salt content in Picual table olives fermentation is feasible, while its inoculation could potentially lead to the production of an added value product.

Initially, the evolution of microbial communities was studied using NGS analysis. The analysis of 16S rRNA metagenomic has fundamentally enhanced our knowledge regarding bacterial communities during Picual olive fermentation. However, some concerns regarding this analysis have been noted. For instance, a potential preferential amplification could impact the number of OTUs, leading discordance to the real abundance of microbes (Ercolini, 2013). Furthermore, the discrimination between living and dead cells is not feasible, leading to a miscalculation of the real microbiota, presented in a habitat (Emerson et al., 2017). Nevertheless, in the present study, using this culture-independent method, an in-depth study of bacterial consortia was achieved up to species level, allowing to a better understanding of this complex matrix, as table olives fermentation is supposed to be. Our findings show that different microbiome profiles observed in the middle (day 60) and at the end of the process, which are closely linked to the inoculation with the starter culture. More specifically, the dominance of *L. plantarum* group in inoculated treatments was profound, indicating that starter culture withstands the competition with the natural microflora, while is not affected by high salt concentration and predominates in a short time. This is in agreement with the literature, as Pino et al. (2019) reported the dominance of the starter inoculation, while other studies highlighted the successful inoculation during several olives’ fermentations, as well (Panagou et al., 2008; Tataridou and Kotzekidou, 2015; Chranioti et al., 2018; Pino et al., 2018). Oppositely, *L. brantae* and *L. parakefiri* predominated in non-inoculated S1. To our knowledge, this is the first study reporting these two LAB as predominant species in table olives fermentation. Rodríguez-Gómez et al. (2017), indicated the predominance of *Lactobacillus* genus in fresh and cured green *Alorena* table olives at the end of fermentation. Cocolin et al. (2013) also found that after three months of fermentation *Lactobacillus* spp. was the main bacterial population present. Both studies are in line with the present study. However, the microbiome profile of the second non-inoculated treatment S4 was unexpected. The different profiles between non-inoculated treatments (S1 and S4) were linked with the olives’ technology (whole fruit vs cracked). At the end of the process, S4 had the highest biodiversity, with a co-existing pool of microorganisms. Notably, apart from the presence of LAB (*L. plantarum group, L. manihotivorans*, etc.), there is also a worrying presence of *Enterobacteriaceae*, *Chlorobacteria*, and other environmental or fruit flesh originated microorganisms, which were not detected with culture-based approaches and reported for the first time, indicating the usefulness of HTS analysis. Furthermore, this finding strongly supports the hypothesis that starter culture use is fundamental in table olives fermentation, to ensure safety and appropriate microbial succession. In a previous study, Medina et al. (2016), using pyrosequencing analysis, reported the presence of undesirable *Celerinatantimonas*, *Pseudomonas*, and *Propionibacterium* as the most abundant genera detected in traditional industrially fermented fruits. Moreover, the lower relative abundance of *L. plantarum* group in S3 treatment indicates high competition between starter culture inoculum and indigenous microflora, which were probably able to grow under lower NaCl content, as we are referring to salt intolerant species like *L. manihotivorans* and *L. parakefiri*. Indeed, in previous study, it has been reported a potential competition between starter and indigenous microflora (Chranioti et al., 2018). Finally, based on literature findings, the determination of microbial communities of table olives fermentation is in early research stages. Thus, HTS analysis could be a reliable tool to solve this aspect. However, the monitoring of microbiome profile during olives fermentation of different years, origin and production with reduced NaCl content is strongly recommended. This will further ensure the stability of the fermentation using LAB starter. Furthermore, a study aiming to associate the existed microbiome with volatile compounds secretion during olives fermentation would also be of great importance. Indeed, no such information is available up to now.

The different microbial communities between treatments, led to diverse microbial populations and biochemical attributes, as revealed from our results. In particular, the higher population levels of LAB in inoculated treatments caused higher reduction of pH values and higher levels of TA, which were lower than the limits proposed by International Olive Council [IOC] (2004) (4.3 and 0.4, respectively), while the respective values of non-inoculated treatments were close to those limits. The latter could be attributed to the higher production of lactic acid in inoculated samples, as revealed by HPLC analysis. The increased acidic conditions in inoculated treatments led to the elimination of *Enterobacteriaceae* and *coliforms* in a shorter time, as well. It is crucial to mention that in S4 non-inoculated sample, these undesirable microorganisms were detectable for a more extended period (60 days), enhancing the hypothesis that inoculation is mandatory to ensure the safety and succession of fermentation. The profound positive effect of LAB starter in those aspects is in agreement with the literature (Papadelli et al., 2015; Pino et al., 2018).

Furthermore, the high acidic environment in inoculated treatments increased electrical conductivity. The proportion of pH and conductivity has already been reported (Liebeherr, 2006). This is in line with our previous study, that indicated a correlation between pH, TA and electrical conductivity (Anagnostopoulos et al., 2020). From the same study, this method has been proposed for the first time as an alternative tool for table olives fermentation monitoring. The findings from the present study are strongly enhance this hypothesis. Notably, this method has been used in the fermentation of other products, as well. Particularly, Cais-Sokolińska (2017) reported a clear association between pH and conductivity during mixed coagulation of milk. Regarding the water potential, it is obvious that osmosis pressure in treatments with lower NaCl content was higher than the others, allowing a faster diffusion of fruit’s elements (oleuropein, sugars, etc.) to the brines. In line with this observation, Papadelli et al. (2015) noted that the deceleration in the diffusion of soluble compounds from fruit to brine is closely linked with high NaCl concentration. Furthermore, this agrees with our previous study (Anagnostopoulos et al., 2020), where the use of water potential has been proposed as a tool for soluble component kinetic estimations of table olives during the fermentation process.

Concerning organic acids, as previously mentioned, lactic was the most abundant acid in all treatments, while its levels were higher in inoculated treatments. This is closely related to the different dominant microbiota between non-inoculated and started samples, leading to higher homofermentative metabolism (Papadelli et al., 2015). Furthermore, the presence of acetic acid, which could be attributed to yeast activity, is noteworthy (Bleve et al., 2014; Bonatsou et al., 2018). However, the contribution of LAB in the production of this acid could also be taken into consideration. Potentially heterofermentative LAB, such as *L. manihotivorans* (Salvetti et al., 2012) and *L. brantae* (Volokhov et al., 2012), may generate acetic acid from fermentable material under particular conditions of environmental stress as well as from the metabolism of citric acid (Laëtitia et al., 2014). Finally, the presence of succinic acid only in inoculated treatments, could be related to the microbial conversion of citric acid to succinic, via a potential shift from homo- to heterofermentative metabolism of the starter culture (Papadelli et al., 2015).

Moreover, inoculated treatments were richer in antioxidant capacity at the end of fermentation, although during fermentation a high impact of olives technology (cracked and whole fruit) was recorded. However, one of the most promising findings in this study is the faster degradation of oleuropein in inoculated treatments; thus, faster olives debittering; resulting in parallel to the production of higher levels of hydroxytyrosol. This finding confirms that the enzymatic activity of the starter culture was higher than the respective of indigenous microflora, affecting the secoiridoid glucosides and their aglycon derivatives (Perpetuini et al., 2018). In agreement with our results, Othman et al. (2009) identified hydroxytyrosol as the main simple phenolic compound found in the brine of inoculated Tunisian black olives. The accumulation of hydroxytyrosol in the brine, as the main simple phenolic compound of olives of Hojiblanca cultivar at the end of fermentation storage, was also confirmed (Pistarino et al., 2013). The appearance of this compound in the brine is mainly related to the hydrolysis of oleuropein (Kiai and Hafidi, 2014). Hydroxytyrosol is an essential phenylethanoid compound, exhibiting important antioxidant activity (Pistarino et al., 2013). It is considered the main marker for the estimation of oleuropein degradation, as well as for the diffusion of phenols from fruits to brines (Randazzo et al., 2011).

Texture and color are playing a major role in consumers acceptance (Luckett, 2016). Since in the present study, no differences were found in texture and color parameters, between non-inoculated and inoculated treatments, we can conclude that the reduction of sodium chloride is advised. Furthermore, the later could be supported by the fact that the starter driven olives were also highly appreciated by the panelists, as indicated by the highest scores in general acceptance. Based on sensory scores, the major differences found were the higher acidity and lower remaining bitterness, while no differences recorded in sensorial texture. All those aspects could be, therefore attributed to the added starter culture, which is in line with the literature (Chranioti et al., 2018).

## Conclusion

Lactic acid bacteria inoculation has been successfully applied to control the fermentation process of Picual table olives, even in lower NaCl concentration, regardless olives technology, affecting their microbial growth, the composition of microbial communities and their biochemical profile. It seems that the time of fermentation was shortened, as the olives debittering was accelerated, while the process was standardized and the final products were improved regarding their functional properties, as well as their organoleptic characteristics, as confirmed by sensory analysis. Inoculation was also useful for faster elimination of possible contaminants and undesired microbial groups, leading to a more safe and controlled process. Overall, the findings of the present study are very promising, enhancing the significant contribution of starter culture, raising in parallel the possibility to produce table olives with reduced salt content.

Last but not least, HTS analysis can fill in the limitations of culture-dependent methodologies. The microbiome during Picual table olives fermentation, that could not be identified with classical molecular methods, is now surfaced. New bacterial species have been detected for the first time using NGS, indicating the extreme need of such works, to improve our knowledge regarding microbiota formation during table olives fermentation. Consequently, further studies are also necessary to determine the influence of these new microbial species on the sensorial characteristics of table olives.

## Data Availability Statement

The datasets presented in this study can be found in online repositories. The names of the repository/repositories and accession number(s) can be found in the article/Supplementary Material.

## Ethics Statement

This study involves the study of fermented olive fruits (Picual olives) with and without the addition of a commercially available starter culture of *L. plantarum*. For the purposes of the study a tasting panel provided its expert knowledge. The Cyprus University of Technology Ethics Committee and its guidelines do not require any review process or approval. National legislation and National Bioethics Committee did not require the study to be reviewed or approved as the methodology and approaches followed, did not fall into any current National or European Union legislation.

## Author Contributions

DA performed experimental design, data curation, formal analysis, investigation, methodology, visualization, and wrote the manuscript. EK performed data curation, formal analysis, investigation, methodology, and revised the manuscript. DT performed experimental design, supervised the project, and revised the manuscript.

## Conflict of Interest

The authors declare that the research was conducted in the absence of any commercial or financial relationships that could be construed as a potential conflict of interest.

## Footnotes

1 http://viceroy.eeb.uconn.edu/estimates/index.html

## Abbreviations

CoNS: coagulase-negative staphylococci
DPPH: 2,2-diphenyl-1-picrylhydrazyl
F-C: Folin-Ciocalteu
GAE: gallic acid equivalent
HPLC: high pressure liquid chromatography
HTS: high-throughput sequencing
LAB: lactic acid bacteria
LSD: Least Significant Difference
MRS: De Man-Rogosa-Sharpe agar
MSA: Mannitol Salt Agar
NCBI: National Centre for Biotechnology Information
NGS: next generation sequencing
OTU: operational taxonomic unit
PCA: Plate Count Agar
PCA: principal components analysis
rDNA: ribosomal DNA
RDP: Ribosomal Database Project
TA: titratable acidity
TEAC: Trolox equivalent antioxidant capacity
TVC: total viable count
VRBGA: Violet Red Bile Glucose Agar
VRBLA: Violet Red Bile Lactose Agar.
