# Supplementary material for: Evolution of Bacterial Communities, Physicochemical Changes and Sensorial Attributes of Natural Whole and Cracked Picual Table Olives During Spontaneous and Inoculated Fermentation
Source: Front Microbiol. 2020 May 29;11:1128. doi: 10.3389/fmicb.2020.01128 (PMC7273852; doi:10.3389/fmicb.2020.01128)
Supplement: Supplementary file 1 [file Image_1.pdf]

Supplementary Material

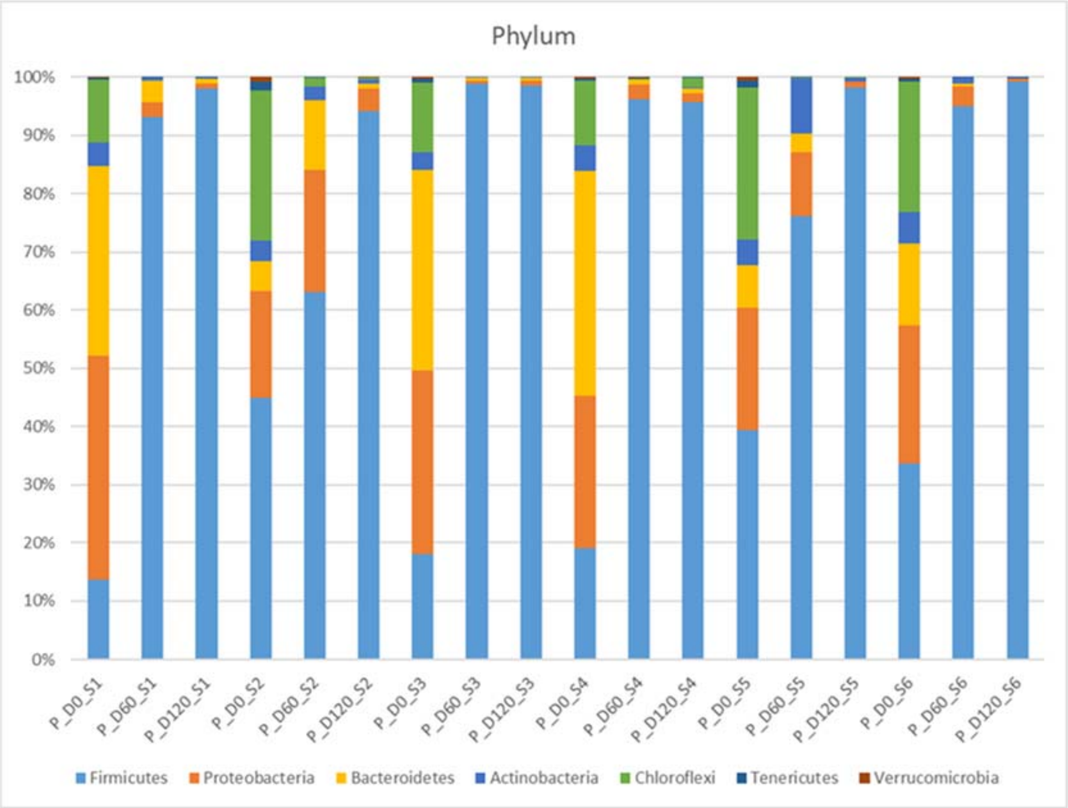

Supplementary Figure 1. Relative abundance of bacterial phyla in the different treatments assayed (S1-S6), obtained through metagenetic analysis of 16S rRNA gene at the initial (D0), middle (D60) and the end (D120) of fermentation process.
